# Psychedelic-Informed Approaches in Forensic Psychiatry: A Systematic Review of Therapeutic Promise, Risk, and Feasibility Within UK Services

**DOI:** 10.1192/bjo.2026.11216

**Published:** 2026-06-30

**Authors:** Sunandan Banerjee

**Affiliations:** Black Country Healthcare NHS Foundation Trust, Birmingham, United Kingdom

## Abstract

**Aims:**

To systematically review the literature on psychedelic-related mechanisms relevant to forensic psychiatry, examining potential therapeutic benefits linked to reduction in crime-related behaviour, associated clinical and legal risks, and the practical considerations required were such approaches ever to be explored within UK forensic mental health services.

**Methods:**

A systematic literature search was conducted in PubMed and PsycINFO in accordance with PRISMA principles. Peer-reviewed publications were screened, including randomised controlled trials, observational and qualitative studies, mechanistic and neurobiological research, reviews, and translational papers relating to psychedelic-assisted therapies. Additional literature addressing trauma, substance misuse, offending behaviour, psychosis risk, forensic rehabilitation, capacity, consent, and criminal responsibility was included. Given heterogeneity in populations, interventions, and outcomes, findings were synthesised using a structured narrative and thematic approach, interpreted within the context of UK forensic psychiatric practice and legal frameworks.

**Results:**

The literature identified several mechanisms with potential indirect relevance to forensic psychiatry. These included enhanced trauma and emotional processing, increased psychological flexibility, disruption of rigid self-concepts and offence-supportive beliefs, improved empathy and perspective-taking, and reductions in substance misuse–factors associated with offending behaviour and recidivism. Such mechanisms are theoretically relevant to offence-related psychotherapy, substance misuse treatment, and rehabilitative interventions within secure services.

However, substantial and consistent risks were also identified, particularly precipitation or exacerbation of psychosis, affective instability, behavioural disinhibition, impaired judgement, and significant challenges in assessing capacity and criminal responsibility during altered states of consciousness. Practical analysis indicated that any hypothetical consideration would require extremely stringent patient selection excluding psychotic disorders, delivery within highly specialised and non-coercive settings, close integration with established psychotherapeutic models, and robust safeguards aligned with the Mental Health Act, Mental Capacity Act, and the forensic mandate of public protection. No evidence supports routine or near-term clinical use in forensic populations.

**Conclusion:**

This systematic review highlights a complex balance between theoretical therapeutic promise and significant clinical, ethical, and legal risks associated with psychedelic-informed approaches in forensic psychiatry. While direct implementation is currently inappropriate within UK forensic services, critical engagement with this literature offers valuable insights into mechanisms of psychological change, rehabilitation, and risk formulation. Any future consideration would require exceptional safeguards, specialist expertise, and a clear ethical mandate centred on patient welfare, legal responsibility, and public safety.

